# The Effect of Prenatal Stress, Proxied by Marital and Paternity Status, on the Risk of Preterm Birth

**DOI:** 10.3390/ijerph16020273

**Published:** 2019-01-18

**Authors:** Anna Merklinger-Gruchala, Maria Kapiszewska

**Affiliations:** Faculty of Medicine and Health Sciences, Andrzej Frycz Modrzewski Krakow University, 30-705 Krakow, Poland; maria.kapiszewska@gmail.com

**Keywords:** women’s health, reproductive health, maternal health, paternal involvement, paternal support, preterm birth, parity, marital status, paternity, birth registry, prenatal stress

## Abstract

Uncertainty and insecurity in the relationship between the mother and father of a child are responsible for heightened maternal stress, which can lead to preterm birth (PTB). Different intensities of prenatal stress (proxied by four levels of marital status linked with the presence or absence of paternal data on birth records) were defined as the Marital-Father Data index. We assessed the impact of those varying intensities of prenatal stress on PTB with respect to parity among a group of Polish mothers residing in Krakow (*N* = 87,916). We found a pattern across the adjusted risk ratios (RR) of preterm birth that ordered these estimates in an increasing trend towards higher risk, beginning with the group of married mothers with father data present (baseline), through the groups of legitimizing marriages—married after conception with father data present (RR = 1.1; 95% Confidence Intervals (CI) 1.0–1.2) and unmarried mothers with father data present (RR = 1.3; 95% CI 1.2–1.5) to the group of unmarried mothers with father data absent (RR = 1.9; 95% CI 1.7–2.2). The adjusted p for the linear trend between Marital-Father Data index and PTB was less than 0.001. The adjusted effect of perceived prenatal stress differed with respect to parity (confirmed by statistically significant interactions between Marital-Father Data index levels and parity), with a higher magnitude of this effect noted among multiparous versus primiparous women. Low paternal involvement and support during pregnancy may negatively affect PTB risk and this effect may differ in relation to parity status. More attention should be paid to maternal pregnancy stress, especially of multiparous mothers, to decrease the risk of unfavorable birth outcomes.

## 1. Introduction

Pregnancy and childbirth are considered happy and joyful life events and usually induce positive emotions. However, under certain circumstances the pregnancy itself can become a stressful and difficult life experience. Psychological science on pregnancy indicates that the consequences of prolonged stress include adverse psychological and physical health effects as well as an increased risk of preterm birth [1,2,3,4,5,6]. Moreover, the current evidence points to pregnancy anxiety as a key risk factor in the etiology of preterm birth. Pregnant women’s vulnerability to stress can be enhanced by such factors as a lack of emotional stability, lack of safety guarantees, uncertainty about the future, having a poor relationship with or weak support from a partner, single status, low education level, financial hardship, young maternal age, having many children in the household, and lack of adequate social support system [7,8,9,10,11,12,13,14]. These stressors may act separately or in an additive way exceeding the adaptive capacity of pregnant women [15]. Uncertainty and insecurity in the relationship between the mother and father of a child (higher among unmarried than married ones) seems to be mostly responsible for heightened maternal stress [6] leading to preterm birth [5,16]. This could imply that pregnancy-specific psychological stress is an independent predictor of adverse pregnancy outcomes [17,18]. Therefore, among unmarried women, the father’s acknowledgment of paternity will also play a significant stress-reducing role [19]. In particular, an unexpected or unwanted pregnancy, especially outside of marriage, is one of the most stressful long-term experiences a woman may have in her life [20,21]. Doubt about the identity of the father of a child, or about the reaction of the women’s family or the father’s family to the news of pregnancy, can generate additional stress. Stress may be particularly devastating when the father doesn’t acknowledge paternity [22,23]. A woman omitting the father’s name on the birth certificate suggests that her relationship with him may be of short duration or characterized by low paternity confidence [22,24], or that the mother does not know who the father of her child is or does not want to identify him for many different reasons. 

The psychological state of mothers who are unmarried but do include the father’s name on a child’s birth certificate is expected to be more stable than that of the previously described group. The inclusion of the father’s name may suggest that he has acknowledged that the child is his, that the couple is cohabiting or in transition from non-residential unions at conception to cohabiting unions at birth [25,26]. Nevertheless, the mother being unmarried (vs. married) or in a non-cohabiting romantic relationship with the father (vs. cohabiting with the father) is still associated with an increased risk of adverse pregnancy outcomes, such as PTB and low birth weight [21,27]. Accordingly, the type of involvement between a mother and father during pregnancy may be viewed as a rough proxy for the stress pregnant women are exposed to. It is well documented that in many countries cohabitation is a prerequisite for marriage and correlates with fertility during partnership [26,28,29]. Most pregnant cohabiting women married before the child was born (i.e., so called shut-gun marriage) [25]. However, transitions to cohabitation or marriage among unmarried women between conception and birth depend more on their male partners’ attitudes towards the relationship than their own attitudes [28]. A “legitimizing marriage” [30] or “reinforcement” of the relationship may be experienced by pregnant women as social pressure, and may enhance their anxiety, which should be related to the level of risk of preterm birth. Thus, to evaluate the true effect of marriage on the risk of adverse birth outcomes, it is necessary to distinguish between marriage at the time of conception, and marriage that occurs prior to the first conception. 

Based on the literature showing that married pregnant women tend to report higher levels of social and emotional support than cohabiting women, and that both groups fare better than single women [29], we hypothesized that the level of maternal stress a mother experiences after formalizing her relationship with the father of her child is lower than the level of stress she experiences when the couple only cohabits, even if the father’s name is on the child’s birth certificate. 

The importance of the father’s presence and support seems to be more significant for mothers of more than one child. More children at home was found to increase partner conflict because caring for a large family is stressful to couples and can strain a marriage [31,32]. Multiparous mothers are more likely to be dissatisfied with their partner’s involvement [33] and report generally lower level of support from any member of their social network, primarily in practical respects, such as baby-sitting [34]. Further, parity is considered a potential factor influencing the risk of preterm birth. It has been found that multiparity may be associated with the risk of medical complications, placental pathologies and preterm birth [35]. Accordingly, we hypothesized that prenatal stress and so the probability of preterm birth for multiparous women should be higher compared to primiparous women. 

Paternity status has always been analyzed in the context of length of gestation and other birth outcomes in terms of the effect of the level of father’s involvement or support [36,37]. In our study we considered paternity status as a detrimental factor on preterm birth risk via its potential to exacerbate maternal stress. We argued that not only this factor but also pregnant women remaining in informal unions or even premarital pregnancy (in married women) may induce a higher level of anxiety and insecurity about their and their child’s future, which in turn leads to adverse pregnancy outcomes as compared with traditional marriage. We hypothesized that the size of preterm birth risk reflects the intensity of maternal stress related to the mother’s level of insecurity. Since the level of uncertainty is related both to the psychosocial status of pregnant women and to a large extent to the father’s financial support, it is quite likely that parity affects pregnancy outcomes more strongly than in the case of mothers who have more than one child. Thus, we assume that multiparous women are more likely to experience an elevated level of psychological distress during pregnancy resulting in greater increase in PTB as compared to the primiparous level. Therefore, we constructed the Maternal-Father Data index with four study groups comprising women for whom the father’s data were present or absent on the birth record (paternity status) together with women of four marital statuses (which serves as a proxy for the intensity of maternal psychosocial stress evoked by the perception of insecurity): (1) married before conception (direct family forming), with father data present (MBC-FDP), (2) married after conception (legitimizing marriages) with father data present (MAC-FDP), (3) unmarried with father data present (UM-FDP), and (4) unmarried with father data absent (UM-FDA).

## 2. Materials and Methods

According to the Polish legislation during the study period, the mother’s husband is presumed to be her child’s father. Therefore, no missing paternal data is observed in the birth records among married woman during the study period, whilst for unmarried couples, a father must sign an acknowledgment or have paternity established in court to list his data on the birth certificate [38]. While the father’s data (such as his age, education, and employment) is present in a birth record it advocates a closer relationship between the mother and the acknowledged or the court-established father. Such fathers are more likely to be involved in the support and care of the pregnant woman. In contrast, omitting the father’s name on the birth record by the mother may suggest that their relationship is of short duration, generally of low paternity confidence, or that the mother does not know who the father of her child is or does not want to identify him for many different reasons. The presence or absence of father’s data on the birth record (paternity status) together with maternal marital status were the variables of primary interest in our study. We constructed the Marital-Father Data index with four levels, which serve as a proxy for the gradation of maternal psychosocial stress evoked by low paternal support: (1) married before conception (direct family forming), with father data present (MBC-FDP) (2) married after conception (legitimizing marriages) with father data present (MAC-FDP), (3) unmarried with father data present (UM-FDP), and (4) unmarried with father data absent (UM-FDA). The father’s data was considered absent from the birth record when all fields provided for the father’s data, i.e., his age, education and employment were blank. 

The cohort included singleton life born infants, born after gestations lasting between 25 and 42 weeks from over the 14 years period between 1 October 1995 and the end of December 2009 to mothers whose residence at the time of infant’s birth was the city of Krakow. Multiple gestation and stillbirths were excluded from the cohort. We obtained data from the birth registry (Central Statistical Office in Poland). Data included month and year of birth, birth weight (in grams), infant’s sex, maternal age (in years), gestational age (in weeks), parity, maternal education (primary, lower secondary, basic vocational, upper general or specialized secondary and academic education), maternal employment status (employed vs. not employed) and maternal marital status (married vs. unmarried, that is single, widow, divorced or in separation). Paternal data, if present, comprised age (in years), education (primary, lower secondary, basic vocational, upper general or specialized secondary and academic education) and employment status (employed vs. not employed). Preterm birth (PTB) was defined as birth before 37 completed weeks of gestation, and all infants with gestational age between ≥37 to 42 weeks were classified as term births (TB). 

Of 88,474 singleton births we excluded stillbirths (*n* = 387), born before 25 and after 42 weeks of gestation (*n* = 152), those with unknown gestational age (*n* = 12), those with extremely high or low birth weight for a given gestational age (*n* = 5), and with extremely high maternal age (*n* = 2). These exclusions left *N* = 87,916 eligible births, and among them *n* = 4306 (4.9%) newborns was found to be born preterm (PTB).

### Statistical Analysis

In order to identify potential confounders the distribution of several known risk factors for PTB across different exposure categories was examined. Mothers were divided into two categories according to parity: primiparous (reference category) and multiparous, i.e. women with two or more births. Maternal and paternal employment status was divided into two groups: employed vs. not employed. Maternal and paternal education was stratified into two levels: lower education (secondary education without final high school exams, such as: primary education, lower secondary or basic vocational education) and higher education (passed at least final high school exams, which corresponds to British A-levels, such as: upper general or specialized secondary and academic education). Because of strong association between education and employment status, we calculated indicator of employment and education (called “Emp & Edu”) with four groups: “Not Employed-Low Education” (NE-LE), “Not Employed-High Education” (NE-HE), “Employed-Low Education” (E-LE) and “Employed-High Education” (E-HE), with the last one being the reference group. 

The distribution of characteristics of births among primiparous and multiparous mothers and among Marital-Father Data index levels were tested with one-way ANOVA for continuous variables and Chi-square test for categorical variables. In order to estimate risk ratios (RR) with 95% confidence intervals (CI) for the association between Marital-Father Data index and PTB, modified Poisson regression with a robust error variance was performed. Poisson regression was used because the log-binomial models did not converge to provide parameter estimates. Firstly, only the Marital- Father Data index (with four levels) was entered as a categorical predictor of PTB (crude effect). Next, we built the adjusted model by entering both social factor: Emp & Edu groups and demographic confounders such as: maternal age (continuous), sex of the child, and parity. Additionally, adjusted linear trend was tested by using the Wald statistics in which Marital-Father Data index with 4 levels was treated as a single ordinal variable in adjusted model.

Subsequently, we attempted to assess whether the effect of Marital-Father Data index differs between primiparous and multiparous mothers. Therefore we conducted the same procedure, after stratification for parity. The effect modification by parity, was further tested by adding the product term (Marital-Father Data index x parity) into the full model. The effect modification term expresses here by how much the effect of Marital-Father Data index differs between parity strata. In addition, the visualization of interaction between Marital-Father Data index and parity and its impact on PTB after adjustment to confounders were plotted. An alpha level of 0.05 was taken as the threshold for statistical significance.

## 3. Results

Among the study cohort of 87,916 births, father’s age, education and employment were recorded for *n* = 84,047 (95.6%), *n* = 83,866 (95.4%), *n* = 83,828 (95.4%) neonates (respectively), whilst for *n* = 3806 (4.3%) all of the above three categories were left missing. The univariate analysis suggested that the risk ratio for PTB was significantly associated with maternal age, sex of the offspring, parity, maternal education, and maternal marital and employment status (Table 1). 

Mothers who had male offspring had a 14% higher risk of PTB than those who gave birth to a girl. For every additional year more of maternal age, the risk of PTB increased (RR = 1.02, 95% CI 1.02–1.03). Delivering second or subsequent child, was associated with 9% higher risk of preterm birth than among primiparous. Unmarried women had a 60% higher risk of preterm birth than married women. 

Having a lower education level was associated with 54% elevated risk of PTB in comparison to higher education levels, whilst being unemployed vs. employed increase the risk by 29%. When analyzing maternal educational and employment status together, women who simultaneously had lower education and no employment, had 75% higher risk of PTB than women with both employment and higher educational level (Table 1). The parity strata differed according to maternal age, sex of the child, maternal marital, employment and educational status, and Emp & Edu indicator and birth status (being born term or preterm, Table 2). 

For the mothers exposed to different levels of Marital-Father Data index the characteristics varied according to maternal age, maternal marital, employment and educational status, Emp & Edu indicator, but not sex of the child (Table 3).

Both univariate and multivariate analyses conducted among the entire cohort revealed that mothers belong to MAC-FDP group did not differ significantly from the reference group (MBC-FDP). While the UM-FDP group of women had elevated risk of PTB, both before and after standardization for maternal-infant characteristics. Whereas the last level of Marital-Father Data index (UM-FDA) was associated with almost two times increased risk of PTB in comparison to MBC-FDP both in crude and adjusted model (Table 4). 

**Figure 1:**
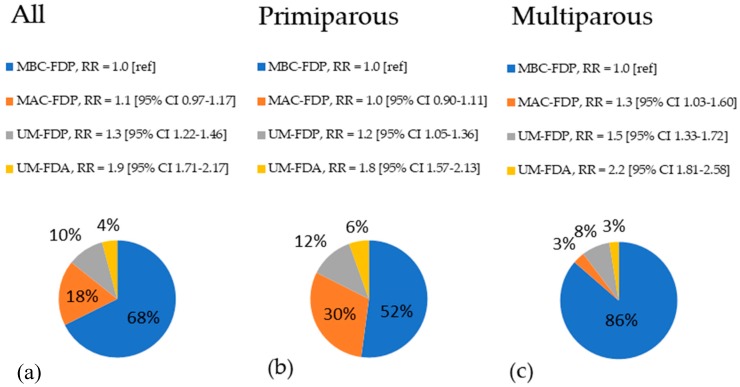
Pie charts of frequencies of Marital-Father Data groups in the entire group of mothers (**a**) and separately among primiparous (**b**) and multiparous (**c**) together with adjusted risk ratios of preterm birth, associated with different types of mother-father relationship (proxied by Marital-Father Data groups), calculated with reference to MBC-FDP group.

Adjusted p for linear trend between Marital-Father Data index and PTB was less than 0.001. After stratification for parity, the same pattern were observed among primiparous women. Among the strata of multiparous mothers, all of the subsequent levels of the Marital-Father Data index differ significantly from the baseline level, and the magnitude of association was stronger than among primiparous. Specifically, the MAC-FDP, UM-FDP and UM-FDA groups of multiparous mothers were associated with 1.29, 1.51 and 2.16 times higher risk of PTB (respectively) in comparison to MBC-FDP after adjustment for confounders, with an adjusted p for linear trend of less than 0.001 (Table 4). Adjusted risk ratios of preterm birth, associated with different types of mother-father relationship, together with frequencies of Marital-Father Data groups, both before and after stratification for parity, are presented on Figure 1.

These different effects of the levels of Marital-Father Data index across the parity strata was confirmed by the statistical interaction (Wald test, *p* < 0.001). The evaluation of effect modification on multiplicative scale indicated that in reference to primiparous mothers belonging to MBC-FDP group, among multiparous mothers from MAC-FDP, the additional multiplicative increase in risk of PTB attributable to being multiparous beyond the effect of belonging to this group alone, was 33% (RR for interaction = 1.33, 95% CI 1.05–1.69;). The corresponding additional multiplicative increase in risk of PTB among the group of multiparous UM-FDP was 36% (RR for interaction = 1.36, 95% CI 1.14–1.63). Whilst belonging to UM-FDA group of multiparous mothers, the additional multiplicative increase in risk of PTB attributable to being multiparous beyond the effect of belonging to this group alone was even 37% (RR for interaction = 1.37, 95% CI 1.10–1.71).

**Figure 2:**
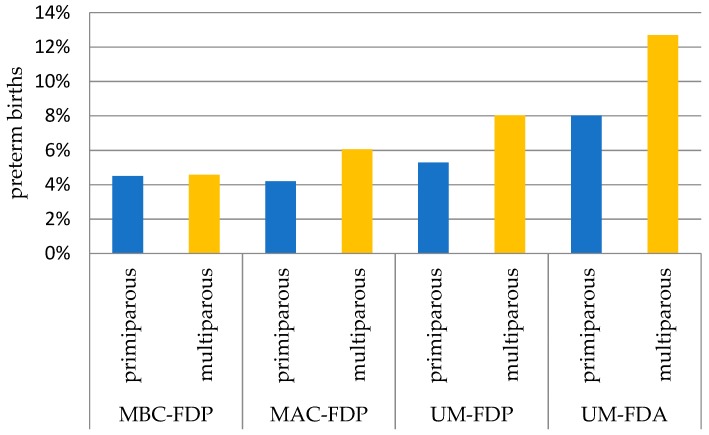
Preterm births by Marital-Father Data index (as a proxy of maternal psychosocial stress) and parity. MBC-FDP: married before conception & father data present; MAC-FDP: married after conception & father data present; UM-FDP: unmarried & father data present; UM-FDA: unmarried & father data absent.

In order to present the full picture of interaction between Marital-Father Data index and parity we also provided the estimates of parity effect alone after stratification for Marital-Father Data index groups (Appendix A, Table A1). Among MBC-FDP group, being multiparous vs. primiparous mother was associated with decreased risk of PTB by 22% (RR = 0.78, 95% CI 0.72–0.85) after adjustment for confounders. Whilst among other groups of Marital-Father Data index, i.e., MAC-FDP, UM-FDP and UM-FDA, being multiparous v. primiparous mother was associated with increased risk of PTB by 28% (OR = 1.28, 95% CI 0.99–1.65), 21% (RR = 1.21, 95% CI 1.01–1.46) and 37% (RR = 1.37, 95% CI 1.07–1.74), respectively, after adjustment RR confounders. Being multiparous v. primiparous decreased the risk of PTB among entire group of women by 10% (RR = 0.90, 95% CI 0.84–0.97). To visualize the interaction between Marital-Father Data index and parity we plotted the prevalence of PTB in Figure 2. The interaction effect is reflected by the increasing parity gap between primiparous and multiparous mothers as moving from the group of married mothers with father data present (MBC-FDP) through the groups of legitimizing marriages (married after conception with father data present: MAC-FDP) and unmarried mothers with father data present (UM-FDP) to the group of unmarried mothers with father data absent (UM-FDA).

## 4. Discussion

We hypothesized that being an unmarried pregnant mother is more closely associated with elevated risk of preterm birth than being a married mother, probably due to enhanced psychosocial stress [39,40]. Moreover, to be unmarried and rejected by the father of a child, or not knowing his identity (UM-FDA), is related to even higher risk of preterm birth than being unmarried with acknowledgement of paternity (UM-FDP). Regarding marital status, we assumed that getting married after conception (MAC) [41] is more stressful than getting married before conception (MBC). Thus, we expected that these four types of relationship between mother and father (described as the “Marital-Father Data index”) represent different intensities of prenatal stress. We predicted that these varying levels of prenatal stress would result in gradations of preterm birth risk. Our prediction was supported by the data: the analyses showed the existence of a pattern across the risk ratios which ordered them in an increasing trend towards higher risk of preterm birth, moving from the group of married mothers with father data present (baseline), through the groups of legitimizing marriages (married after conception with father data present: RR = 1.06; 95% CI 1.0–1.2) and unmarried mothers with father data present (RR = 1.33; 95% CI 1.2–1.5), to the group of unmarried mothers with father data absent (RR = 1.93; 95% CI 1.7–2.2). This was confirmed by a statistically significant p for trend analyses conducted both among the entire group of mothers and separately among primiparous and multiparous mothers. The stratification for parity revealed the same pattern of association between the Marital-Father Data index and PTB for both primiparous and multiparous mothers, as was observed in the entire group of women. However, among the group of multiparous mothers, the relationship between PTB and the Marital-Father Data index levels seemed to be steeper than among primiparous mothers. The harmful effect of the father’s absence, while concomitant with being unmarried on the risk of PTB (i.e. belonging to the UM-FDA group), was well illustrated by a two-fold increase in the risk of PTB as compared to the reference group (MBC-FDA) among the entire group of pregnant women. However, the voluntary establishment of legal paternity in unmarried couples (UM-FDP) enhanced the risk only by 33%, as compared to MBC-FDP. In order to determine the effect of the father’s absence alone in unmarried mothers, we reran the same model and set UM-FDP as the reference group. In this case, the adjusted risk of PTB were 45% higher for UM-FDA among the entire group of pregnant mothers and this effect did not differ across parity strata (p for this particular interaction term was 0.95). Since it has been shown that the socio-economic status is an important predictor of birth outcome and also of antenatal anxiety [42,43,44,45,46,47,48,49,50], all our analyses were adjusted for maternal employment and education, as well as the mother’s age and the sex of the child. There are no empirical studies, to our knowledge, which show the association between marital status and risk of preterm birth with respect to parity. 

Uncertainty about the identity of the father of a child and his (and his family’s) reaction to the news of pregnancy can generate additional stress for unmarried women. It has been found that these women are more likely to have some symptoms of depression and to experience more serious life events during pregnancy [49,50]. In some cases, in the absence of an offer of marriage, single women even resorted to abortion to avoid compromising their futures [51,52]. Furthermore, in such cases it is very likely that pregnancy is unexpected or unwanted [53,54]. It was observed that higher pregnancy anxiety was independently related to unintended pregnancy and this in turn may lead to preterm birth [20,54,55,56].

It is highly likely that the partners of mothers in the UM-FDA group do not believe that the child in question is actually theirs. This, in turn, may explain why these fathers do not want to recognize their relationship with the mother and do not want to establish paternity. This is one of the most stressful long-term experiences a woman may have in her life. A low level, or lack of both emotional and financial paternal support during pregnancy predicts the occurrence of unfavorable perinatal outcomes [57,58,59]. These findings and the results of our study are in line with other well-established research which indicates that general maternal distress and depression during pregnancy, caused by a sense of insecurity and anxiety, negatively affect fetal growth and increase the risk of adverse perinatal outcomes, including preterm birth [16,18,60]. The absence of fathers is consistently related to reproductive outcomes in industrialized countries [61]. The physical and psychological strains associated with pregnancy can be mitigated by emotional support and material resources.

The existing literature confirms that the voluntary establishment of legal paternity by a father can be a proxy for both paternity confidence (a man’s assessment of the likelihood that he is the father of a putative child) and male commitment to investing in offspring, all of which reduces the anxiety of pregnant women [19,22]. To establish legal paternity for unmarried women, in Poland, as in many other countries, a man can voluntarily sign an Acknowledgment of Paternity form, and in such case his name will appear on the birth certificate. This fact has important consequences for both the mother and the child and implies a fathers’ involvement and compliance with child support [24,62]. It was shown that when a partner’s support is effective, that is when it meets a woman’s needs, it correlates well with lower anxiety in pregnancy [63]. This finding suggests not only that the father of the child has acknowledged that the child is his, but also that the couple may be cohabiting, or in transition from a non-residential union at conception to a cohabiting union at birth [25]. The involvement of the father in a mother’s pregnancy and later in the child’s life, even outside marriage, usually serves as an effective buffer that provides multiple sources of financial and care-giving support, therefore reducing the mothers’ stress during pregnancy [64], as compared with a single mother. 

A survey in France showed that the odds ratio of preterm birth among unmarried mothers living without the father was 1.9, whilst for unmarried mothers cohabiting with the father it was 1.6 [65]. Our results confirmed those findings showing that the risk ratio of preterm birth was 1.9 for unmarried mothers with father data absent, whilst for unmarried mothers with father data present the risk ratio was 1.3. Nevertheless, being unmarried (vs. married) or in a non-cohabiting romantic relationship with the father (vs. cohabiting with the father of a baby) is still associated with an increased risk of adverse pregnancy outcomes such as low birth weight [56] and preterm birth [66]. Although cohabitation is becoming more common as a setting for childbearing [67,68,69], in some countries, with highly conservative social and religious norms, a child born outside of marriage is still categorized as illegitimate [70]. Unmarried pregnant women in countries such as Poland, where the vast majority of people declare themselves as Catholic, may be particularly vulnerable to stress. It has been shown that the internalization of Catholic values is so strong that even those who do not declare themselves to be religious, and who are not members of the Catholic Church, seem to be affected by it [71,72]. In addition, cohabitating women may not only suffer social disapproval, but may experience law-related disadvantages due to their status [19]. In more conservative countries, cohabiting couples experience different judicial outcomes in relation to social security and tax, inheritance, and welfare benefit rights [73], as compared with married couples. The lack of legal protections for cohabiting unions also makes it easier for men to disengage after the birth of a child, as compared to more regulated and financially binding marriages; this generates uncertainty for pregnant women in such unions. Married pregnant women tend to report higher levels of social and emotional support than cohabiting women, and both groups are far better off than single women [29,68,69,74,75]. The advantages of marriage in terms of perinatal health and birth outcome as compared to cohabiting unions is well documented [75,76]; however, in the analysis of individual risk factors, it is postulated that social and cultural aspects should also be considered [39]. Our results additionally indicate that when a marriage occurs should also be taken into consideration. Recently, the sequence and timing of partner formalization and childbearing have changed dramatically. A growing proportion of marriages begins with premarital conception [40]. In our study this proportion of marriages is higher than 30%. The question remains of whether the marriage status at the time of birth versus time of conception affects the perinatal outcome. It has been shown that if the first pregnancy occurs in a cohabiting union, the couple is more likely to marry [77]. Since the marriage decision is usually the man’s, and mid-pregnancy marriages (sometimes colloquially referred to as “shotgun marriages”) seem to be relatively fragile [30,41], we decided to treat the sequence of marriage and conception as a stress-related factor. Consequently, the “Marital-Father Data index” that we created includes two forms of marital status: (1) a couple gets married after it learns about a pregnancy (MAC), the so-called “legitimizing marriage” or “reinforced marriage” [30] and (2) the traditional, old-fashioned marriage which occurs prior to the first conception (MBC). 

Contrary to our expectation, the results show that this factor (MBC vs MAC) did not affect the PTB risk when the analysis was conducted among the entire group. Also, the relation between the time of marriage (before or after conception) and PTB risk was not statistically significant for primiparous women. Significantly elevated risk of preterm birth for MAC, as compared to MBC, were observed only among multiparous women (RR = 1.29, 95% CI 1.03–1.60). Formalization of the union during pregnancy may indicate that the couples remain in cohabitation, and this type of relationship preceded the “legitimizing marriage” [25] in both primiparous and multiparous women. However, the higher risk of PTB corresponds closely with the results of a study conducted on parents across Europe and in the United States which showed that unintended pregnancy generates greater stress for multiparous than for primiparous women, especially when it occurs outside of marriage [69]. According to other research on parity differences, the chances of “legitimizing marriage” for multiparous women are also lower [25]. This observation seems to be confirmed by our results, showing that the percentage of multiparous women who get married after conception is tenfold smaller than primiparous women (3.3% and 30%, respectively). Some studies claimed that multiparity implies increased social and financial stress for a mother [78], which can result in exacerbated anxiety and insecurity. 

It was shown that multiparous, as compared to primiparous mothers, have a lower rate of obstetric complications [56,79,80,81]. This general conclusion was supported by our finding of diminished risk of PTB for multiparous women, when the entire group of mothers was analyzed. However, when we took into consideration the stratification for the Marital-Father Data index, only among the traditional marriage group (MBC-FDP), being multiparous vs. primiparous, diminished the risk of PTB; however, among all other groups of the Marital-Father Data index, that is MAC-FDP, UM-FDP and UM-FDA, delivering a second or subsequent child was associated with higher risk of preterm birth. Both of these results strongly suggest that pregnancy or even conception outside marriage is more stressful for multiparous women than for primiparous women. This result again strongly supports the hypothesis that a father’s presence is much more important for multiparous mothers, than for primiparous mothers, in terms of the risk of preterm birth. 

The relationship between socioeconomic disadvantage and adverse pregnancy outcome in industrialized countries was systematically reviewed by Blumenshine et al. [48]. They underlined that despite decades of research and increased attention to prevention, socioeconomic disparities in birth outcomes among most population groups remain prevalent and substantial. They emphasized that some socioeconomic factors, in particular occupational class and educational achievement, were more frequently associated with adverse birth outcomes than others. These factors may be linked with unhealthy behaviors, which in turn affect pregnancy outcomes. Additionally, the authors noticed, that this discrepancy may also reflect significant unmeasured socioeconomic and psychological contexts, such as social pressure, cultural factors, or other social experiences, including those indicated by our study. The effect of age on the risk of very preterm birth in teenage multiparous mothers was observed, after controlling for other risk factors [82,83]. 

Our results also seem to be in agreement with those of MacDonald LD et al. [49], who showed that unmarried (versus married) women were on average younger, less educated, of lower social class and in poorer economic circumstances. The social characteristics of pregnant women, their marital status, and whether or not they lived with the father of the child have also been analyzed by others [65,84]. It was established that unmarried women living with the father of their child, similar to unmarried women living alone, were more often younger, having their first baby, had less education and were more likely to be unemployed than married women living with the father. Also, the highest rates of symptoms of anxiety and depression occurred among women with only primary education [50]. Our results not only confirm these observations, but additionally underline the importance of socioeconomic and demographic factors in PTB. It was also highlighted that the stability of a couple’s union is enhanced by maternal economic contributions [85]. In particular, childbirth outside of marriage is strongly associated with social and economic disadvantages [86]. We observed that the risk of PTB in each of the investigated groups were higher after controlling for socioeconomic status as compared to the crude results. We suggest that a combination of socioeconomic position and civil status (Marital-Father Data index) can be interpreted as a proxy of prenatal stress. 

It was postulated that the association between maternal socioeconomic position and adverse pregnancy outcomes, including preterm birth, can be explained by stress-activated hypothalamic-pituitary-adrenal (HPA) responses [87,88,89,90,91,92,93] and also by other biological mechanisms [93,94]. The intensity of the stress/signal is related to the level of corticotropin-releasing hormone (CRH) [95]. CRH during the activation of HPA serves as a mediator between maternal stress and the length of gestation. Cortisol, the primary stress hormone, also helps a fetus to develop and is responsible for triggering the birth process. Cortisol could also contribute to premature labor [96,97,98,99]. Long-term activation of the stress-response system, high-intensity of stressful conditions, and the subsequent overexposure to cortisol and other stress hormones can lead to adverse pregnancy outcomes [100,101]. It has to be kept in mind that psychological stress is experienced subjectively and secretion of stress hormones varies individually. Therefore, one person may secrete higher levels of cortisol than another in the same situation, thus the final effect can be different.

This study was based on birth registry data; therefore, it contains some limitations which should be discussed. Our study took advantage of the fact that for some records the paternal data were missing. We could only assume the reasons for missing partner information. We hypothesized that it may serve as an indicator of paternal involvement. While other explanations are possible, such missing father data has been used elsewhere as a surrogate measure of lack of paternal involvement, for example [23,61,102] and predicted adverse pregnancy outcomes. Another limitation of our study is the lack information on several factors which might affect the risk of preterm birth, such as cohabitation status among unmarried women or acknowledgement of paternity in court. These are important omissions as cohabitation and acknowledgement of paternity are likely to be associated with men’s willingness to invest in children and support them. The lack of information on relationship history, family structure and multipartner fertility may also have affected our results. Unfortunately, our study does not allow us to evaluate the effectiveness of partners’ support in any of the analyzed groups or how this support was perceived by women. Further studies are needed to explore how the mother–father relationship affects the timing of pregnancy and whether it depends on other stressors besides the number of children a mother already has.

## 5. Conclusions

Our study added further support to the hypothesis that lack of paternal involvement may negatively affect perinatal outcomes and confirmed our prediction that this negative impact may differ in relation to the parity status of a mother. For women who already have children, such deprivation of a father’s support may generate greater stress, than for women giving birth to their first child. To verify these predictions, further studies are needed. These studies should evaluate the true effect of marriage on the risk of adverse birth outcomes, i.e. to distinguish between Direct Family-Forming marriages and marriage after co-habitation (referred to as “Post-Cohabitation Family-Forming” by Holland [30]). Unfortunately, our data did not allow us to distinguish between them. 

Drawing on the literature on contemporary changes in demography and family structure, when an increasing number of children are born outside of marriage [65,103,104], it is worth considering the increased risk of unfavorable birth outcomes that may be related to deprivation of the security of pregnant women [105]. Bearing in mind that cohabitation and acceptance of out-of wedlock births have become more common in the twentieth century, legal protections for pregnant women in addition to social or economic support seems to decrease the risk of preterm birth. Moreover, the adverse pregnancy outcomes associated with pregnancy anxiety indicate that adequate assessment of this condition is important and that intervention efforts should be planned to help women in high risk situations to cope with anxiety. It was shown that some stress-reduction strategies, such as organizing prenatal care groups, maternal support, education, empowerment, stress-reduction, and coping strategies were very effective in preventing preterm birth [106]. Our study indicates that more attention should be paid to psychosocial stress of multiparous mothers to reach more positive birth outcomes.

## Figures and Tables

**Table 1:** Characteristics associated with preterm birth (PTB) among singleton, live births.

Characteristics	Estimate/Level	Term Birth	Preterm Birth	Risk Ratio (RR)	−95% CI	+95% CI
Maternal age (cont.)	*n*	83,610	4306	1.02	1.02	1.03
	mean	28.1	28.7			
	std. dev.	5.2	6.0			
Sex of the infant	Boy	42,907	2353	1.14	1.07	1.20
		51.3%	54.6%			
	Girl	40,703	1953			
		48.7%	45.4%			
Parity	Multiparous	37,973	2052	1.09	1.03	1.16
		45.4%	47.7%			
	Primiparous	45,632	2253			
		54.6%	52.3%			
Maternal marital status	Unmarried	11,811	915	1.60	1.49	1.71
		14.1%	21.2%			
	Married	71,799	3391			
		85.9%	78.8%			
Maternal employment	Unemployed	21,414	1330	1.29	1.21	1.37
		25.7%	31.1%			
	Employed	61,918	2946			
		74.3%	68.9%			
Maternal education	Lower	18,365	1298	1.54	1.42	1.61
		22.0%	30.3%			
	Higher	64,967	2979			
		78.0%	69.7%			
Emp & Edu Mother	NE-LE	8718	711	1.75	1.61	1.90
		10.5%	16.6%			
	E-LE	9628	585	1.33	1.21	1.45
		11.6%	13.7%			
	NE-HE	12,679	617	1.07	0.98	1.17
		15.2%	14.4%			
	E-HE	52,253	2360			
		62.7%	55.2%			
Marital-Father Data index	UM-FDA	3449	357	2.07	1.86	2.30
		4.1%	8.3%			
	UM-FDP	8362	558	1.37	1.26	1.50
		10.0%	13.0%			
	MAC-FDP	15,101	688	0.96	0.88	1.04
		18.1%	16.0%			
	MBC-FDP	56,698	2703			
		67.8%	62.8%			

cont.—continuous variable; Emp & Edu—indicator of employment and education; NE-LE—Not Employed and Low Education; NE-HE—Not Employed and High Education; E-LE—Employed and Low Education; E-HE—Employed and High Education; N—number of participants, std.dev.—standard deviation; TB—term births (≥37 and ≤42 weeks of gestation), PTB—preterm births (<37 weeks of gestation); MBC-FDP—married before conception, father data present, MAC-FDP—married after conception, father data present, UM-FDP—unmarried, father data present, UM-FDA—unmarried, father data absent.

**Table 2:** Distribution of characteristics of singleton, live births stratified for parity.

Characteristics	Estimate/Level	Primiparous	Multiparous	*p*	ANOVA/Pearson Chi-square
Maternal age (cont.)	*n*	47,885	40,025	<0.01	F (1, 87,908) = 19,578.0
	mean	26.1	30.6		
	std.dev.	4.6	4.8		
Sex of the infant	Boy	24,818	20,438	0.02	Pearson Chi-square: 5.1; df = 1
		51.8%	51.1%		
	Girl	23,067	19,587		
		48.2%	48.9%		
Maternal marital status	Unmarried	8478	4244	<0.01	Pearson Chi-square: 888.3; df = 1
		17.7%	10.6%		
	Married	39,407	35,781		
		82.3%	89.4%		
Maternal employment	Unemployed	12,007	10,737	<0.01	Pearson Chi-square: 37.7; df = 1
		25.1%	27.0%		
	Employed	35,770	29,090		
		74.9%	73.0%		
Maternal education	Lower	9122	10,540	<0.01	Pearson Chi-square: 677.0; df = 1
		19.1%	26.5%		
	Higher	38,650	29,293		
		80.9%	73.5%		
Emp & Edu Mother	NE-LE	4437	4992	<0.01	Pearson Chi-square: 676.9; df = 3
		9.3%	12.5%		
	E-LE	4678	5534		
		9.8%	13.9%		
	NE-HE	7558	5738		
		15.8%	14.4%		
	E-HE	31,067	23,543		
		65.1%	59.1%		
Birth status	TB	45,632	37,973	<0.01	Pearson Chi-square: 8,3, df = 1
		95.3%	94.9%		
	PTB	2253	2052		
		4.7%	5.1%		

cont.—continuous variable; Emp & Edu—indicator of employment and education; NE-LE—Not Employed and Low Education; NE-HE—Not Employed and High Education; E-LE—Employed and Low Education; E-HE—Employed and High Education; *n*—number of participants, std.dev.—standard deviation; TB—term birth; PTB—preterm birth.

**Table 3:** Distribution of characteristics of singleton, live births stratified for Marital-Father Data index.

Characteristics	Estimate/Level	Marital-Father Data Index				*p*	ANOVA/Pearson Chi-square
		MBC-FDP	MAC-FDP	UM-FDP	UM-FDA		
Maternal age cont.)	*n*	59,401	15,789	8920	3806	<0.01	F(3; 87,912) = 5511.1
	mean	29.5	24.7	26.8	23.9		
	std.dev.	4.48	4.58	6.08	6.45		
Sex of the infant	boy	30,519	8133	4625	1983	0.72	Pearson Chi-square: 1.33; df = 3
		51.4%	51.5%	51.8%	52.1%		
	girl	28,882	7656	4295	1823		
		48.6%	48.5%	48.2%	47.9%		
Parity	multiparous	34,478	1303	3126	1118	<0.01	Pearson Chi-square: 13,392.3; df = 3
		58.0%	8.3%	35.0%	29.4%		
	primiparous	24,921	14,486	5793	2685		
		42.0%	91.7%	65.0%	70.6%		
Maternal marital status	unmarried	0	0	8920	3806	<0.01	Pearson Chi-square: 87,916.0; df = 3
		0.0%	0.0%	100.0%	100.0%		
	married	59,401	15,789	0	0		
		100.0%	100.0%	0.0%	0.0%		
Maternal employment	unemployed	11,795	5280	3400	2269	<0.01	Pearson Chi-square: 4770.8; df = 3
		19.9%	33.5%	38.2%	62.0%		
	employed	47,489	10,496	5490	1389		
		80.1%	66.5%	61.8%	38.0%		
Maternal education	lower	10,166	3909	3272	2316	<0.01	Pearson Chi-square: 5575.9; df = 3
		17.1%	24.8%	36.8%	63.4%		
	higher	49,131	11,857	5619	1339		
		82.9%	75.2%	63.2%	36.6%		
Emp & Edu Mother	NE-LE	3943	1779	1953	1754	<0.01	Pearson Chi-square: 9565.03; df = 9
		6.7%	11.3%	22.0%	48.1%		
	E-LE	6218	2129	1311	555		
		10.5%	13.5%	14.8%	15.2%		
	NE-HE	7847	3497	1442	510		
		13.2%	22.2%	16.2%	14.0%		
	E-HE	41,258	8355	4172	828		
		69.6%	53.0%	47.0%	22.7%		

cont.–continuous variable; Emp & Edu—indicator of employment and education; NE-LE—Not Employed and Low Education; NE-HE—Not Employed and High Education; E-LE—Employed and Low Education; E-HE—Employed and High Education; *n*—number of participants, std.dev.—standard deviation; MBC-FDP—married before co nception, father data present, MAC-FDP—married after conception, father data present, UM-FDP—unmarried, father data present, UM-FDA—unmarried, father data absent.

**Table 4:** The association between the Marital-Father Data index (as a proxy of maternal psychosocial stress) and preterm birth (PTB) before and after stratification for parity, tested by simple and multiple Poisson regression analyses. Crude and adjusted risk ratios (RR) with 95% confidence intervals (CI) for PTB by categories of parity.

	Marital-Father Data Index		RR	RR−95% CI	RR+95% CI	RR	RR−95% CI	RR+95% CI
All			Crude			Adjusted *		
	MBC-FDP	[ref.cat.]	1.00			1.00		
	MAC-FDP		0.96	0.88	1.04	1.06	0.97	1.17
	UM-FDP		1.37	1.26	1.50	1.33	1.22	1.46
	UM-FDA		2.07	1.86	2.30	1.93	1.71	2.17
						*p* for trend < 0.001 #		
Primiparous			Crude			Adjusted **		
	MBC-FDP	[ref.cat.]	1.00			1.00		
	MAC-FDP		0.93	0.85	1.03	1.00	0.90	1.11
	UM-FDP		1.17	1.04	1.33	1.20	1.05	1.36
	UM-FDA		1.78	1.54	2.04	1.83	1.57	2.13
						*p* for trend < 0.001 #		
Multiparous			Crude			Adjusted **		
	MBC-FDP	[ref.cat.]	1.00			1.00		
	MAC-FDP		1.32	1.06	1.64	1.29	1.03	1.60
	UM-FDP		1.75	1.54	1.99	1.51	1.33	1.72
	UM-FDA		2.80	2.39	3.29	2.16	1.81	2.58
						*p* for trend < 0.001 #		

* adjusted for maternal age, Emp & Edu Mother, sex of the child and parity; **adjusted for maternal age, Emp & Edu Mother, and sex of the child; # linear trend was tested by using Wald statistics in which Marital-FatherData index with 4 levels was treated as a single ordinal variable. RR—isk ratio, CI—confidence interval; ref.cat.—reference category, MBC-FDP—married before conception, father data present, MAC-FDP—married after conception, father data present, UM-FDP—unmarried, father data present, UM-FDA—unmarried, father data absent.

## Appendix A

**Table A1:** The association between parity and preterm birth before and after stratification for Marital-Father Data index, tested by simple and multiple Poisson regression analyses. Crude and adjusted risk ratios (RR) with 95% confidence intervals (CI) of PTB by groups of Marital-Father Data index.

	Parity Strata		RR	RR − 95% CI	RR + 95% CI	RR	RR − 95% CI	RR + 95% CI
			Crude			Adjusted *		
All	primiparous	[ref.cat.]	1.00			1.00		
	multiparous		1.09	1.03	1.16	0.90	0.84	0.97
			Crude			Adjusted **		
MBC-FS	primiparous	[ref.cat.]	1.00			1.00		
	multiparous		1.02	0.95	1.10	0.78	0.72	0.85
MAC-FS	primiparous	[ref.cat.]	1.00			1.00		
	multiparous		1.44	1.15	1.81	1.28	0.99	1.65
UM-FS	primiparous	[ref.cat.]	1.00			1.00		
	multiparous		1.52	1.29	1.79	1.21	1.01	1.46
UM-NFS	primiparous	[ref.cat.]	1.00			1.00		
	multiparous		1.61	1.32	1.96	1.37	1.07	1.74

* adjusted for maternal age, employment and education, sex of the child and Marital-Father Data index; ** adjusted for maternal age, employment and education, and sex of the child; RR—risk ratio, CI—confidence interval; ref.cat.—reference category, MBC-FDP—married before conception, father data present, MAC-FDP—married after conception, father data present, UM-FDP—unmarried, father data present, UM-FDA—unmarried, father data absent.
